# Role of Graphene in Surface Plasmon Resonance-Based Biosensors

**DOI:** 10.3390/s24144670

**Published:** 2024-07-18

**Authors:** Talia Tene, Stefano Bellucci, Fabian Arias Arias, Luis Santiago Carrera Almendariz, Ana Gabriela Flores Huilcapi, Cristian Vacacela Gomez

**Affiliations:** 1 Department of Chemistry, Universidad Técnica Particular de Loja, Loja 110160, Ecuador; tbtene@utpl.edu.ec; 2 INFN-Laboratori Nazionali di Frascati, Via E. Fermi 54, I-00044 Frascati, Italy; 3 Facultad de Ciencias, Escuela Superior Politécnica de Chimborazo (ESPOCH), Riobamba 060155, Ecuador; 4 Dipartimento di Chimica e Tecnologie Chimiche, University of Calabria, Via P. Bucci, Cubo 15D, I-87036 Rende, Italy; 5 Facultad de Ciencia e Ingeniería en Alimentos y Biotecnología, Universidad Técnica de Ambato, Ambato 180104, Ecuador

**Keywords:** surface plasmon resonance, graphene, biosensors, sensitivity, specificity, stability

## Abstract

This work explores the transformative role of graphene in enhancing the performance of surface plasmon resonance (SPR)-based biosensors. The motivation for this review stems from the growing interest in the unique properties of graphene, such as high surface area, excellent electrical conductivity, and versatile functionalization capabilities, which offer significant potential to improve the sensitivity, specificity, and stability of SPR biosensors. This review systematically analyzes studies published between 2010 and 2023, covering key metrics of biosensor performance. The findings reveal that the integration of graphene consistently enhances sensitivity. Specificity, although less frequently reported numerically, showed promising results, with high specificity achieved at sub-nanomolar concentrations. Stability enhancements are also significant, attributed to the protective properties of graphene and improved biomolecule adsorption. Future research should focus on mechanistic insights, optimization of integration techniques, practical application testing, scalable fabrication methods, and comprehensive comparative studies. Our findings provide a foundation for future research, aiming to further optimize and harness the unique physical properties of graphene to meet the demands of sensitive, specific, stable, and rapid biosensing in various practical applications.

## 1. Introduction

Surface plasmon resonance (SPR) biosensors are an advanced analytical tool that enables real-time monitoring of molecular interactions without the need for labels [1]. The operational principle of SPR biosensors relies on the excitation of surface plasmons—coherent electron oscillations—at the interface between a metal and a dielectric [2]. When light hits this metal–dielectric interface at a specific angle, it induces these electron oscillations, resulting in a notable reduction in the reflected light intensity at that angle [3]. This angle is highly sensitive to changes in the refractive index near the sensor surface, allowing SPR biosensors to detect the binding of molecules to the surface, thereby providing valuable insights into the concentration and dynamics of the analyte.

SPR biosensors find extensive applications across various fields, including medical diagnostics [4], environmental monitoring [5], food safety [6], and pharmaceutical research [7]. In medical diagnostics, they are employed to detect biomolecules such as proteins, nucleic acids, and small molecules, offering a label-free, real-time analysis of biomolecular interactions. Environmental monitoring utilizes SPR biosensors for the detection of pollutants and toxins, while the food safety sector employs them to identify pathogens and contaminants. Additionally, pharmaceutical research benefits from SPR biosensors in studying drug–receptor interactions and conducting kinetic studies, making them a versatile tool for understanding complex biological and chemical processes.

The efficacy of SPR biosensors is significantly influenced by the materials used in their assembly. The choice of metallic layers and substrates is crucial in determining the sensitivity and stability of the sensor [8]. In this context, gold is the most used metal in SPR biosensors due to its excellent biocompatibility, chemical stability, and robust ability to support surface plasmons [9]. The superior properties of gold provide a strong and stable signal, making it ideal for a wide range of sensing applications [10]. Silver, although possessing higher plasmonic sensitivity compared to gold, is less chemically stable [11]. This makes silver suitable for applications requiring heightened sensitivity, provided protective measures against oxidation are implemented. Other metals like aluminum [12] and copper [13] are less commonly used due to lower biocompatibility and chemical stability, but they can offer unique advantages in specific scenarios.

Glass substrates are widely favored for their transparency and ease of modification, providing a stable and inert platform for the metallic layer [14]. Their compatibility with various surface chemistries enhances their utility in diverse applications. Silicon substrates, on the other hand, are advantageous for integrating electronic and optical components, making them suitable for advanced SPR biosensor designs [15]. Polymers such as polydimethylsiloxane (PDMS) are also used, particularly in flexible and disposable SPR biosensors, owing to their low cost and ease of fabrication [16].

**Figure 1:**
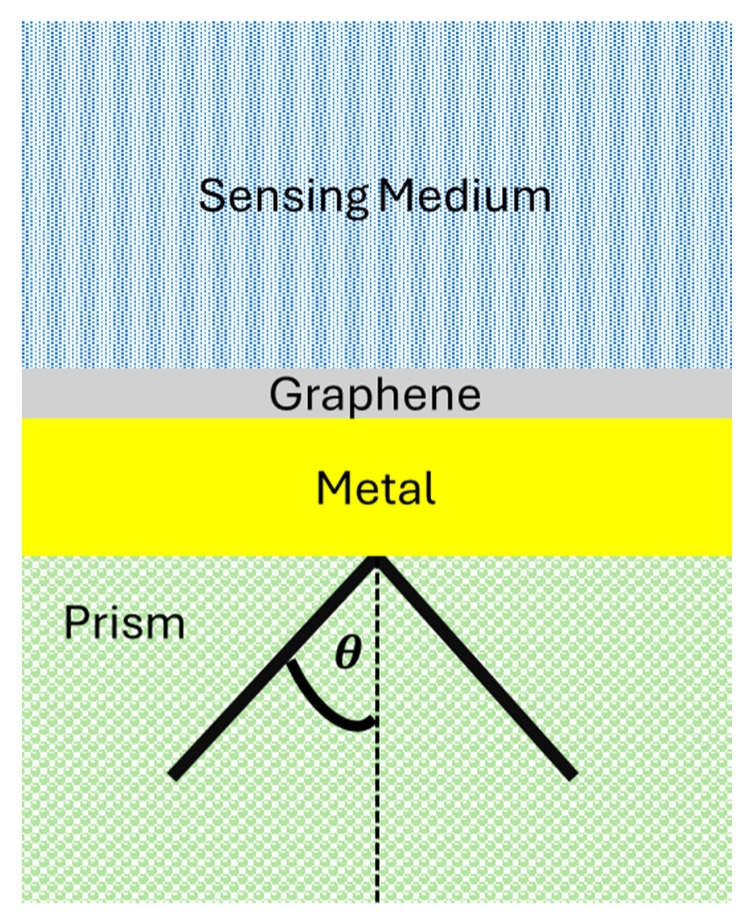
Illustration of a SPR biosensor based on the Kretschmann configuration.

To further enhance the specificity and sensitivity of SPR biosensors, surface functionalization is essential. Self-assembled monolayers (SAMs) of alkanethiols are commonly used to functionalize the gold surface, providing a well-defined and stable interface for immobilizing biomolecules [17]. Polymer brushes can also be grafted onto the metal surface, offering a high-density and flexible environment for biomolecule immobilization [18]. In the last decade, materials like graphene and graphene-derived materials have gained interest due to their excellent electronic properties and large surface area, which can significantly enhance the performance of SPR biosensors [19]. Figure 1 illustrates the use of graphene in SPR biosensors which is based on the Kretschmann configuration [20]. Other works also have focused on advancements in sensor technology using innovative metamaterials [21] and microfibers [22].

Graphene, a single layer of carbon atoms arranged in a two-dimensional honeycomb lattice, has garnered significant attention in various fields due to its outstanding physical and chemical properties [23]. This material exhibits exceptional electronic, mechanical, and thermal properties, making it a versatile material for numerous applications. In the context of SPR biosensors, graphene is known for its excellent electrical conductivity and strong mechanical strength. Furthermore, its electronic properties, such as high carrier mobility and tunable bandgap (e.g., graphene ribbons), make it an ideal material for sensing applications [23]. Additionally, the chemical stability and ability of graphene to undergo surface modifications enable the development of highly specific and sensitive biosensors. Remarkably, graphene can be utilized to enhance the sensitivity and specificity of the sensor [24]. The incorporation of graphene into SPR biosensors can improve detection capabilities by increasing the adsorption of biomolecules onto the sensor surface. This is due to the large surface area of graphene, which provides more active sites for biomolecule binding [25]. Moreover, its excellent electrical conductivity ensures efficient transduction of the biomolecular interactions into measurable signals.

The ability of graphene to support surface plasmons makes it an excellent candidate for SPR biosensor applications [26]. Specifically, when graphene is integrated into the sensor design, it can amplify the plasmonic signals, leading to higher sensitivity. This is particularly useful for detecting low concentrations of analytes, which is crucial in applications such as medical diagnostics [27] and environmental monitoring [28]. Furthermore, the surface of graphene can be functionalized with various biomolecules, such as antibodies, aptamers, or DNA, to achieve high specificity in target detection [29].

On the other hand, the stability of SPR biosensors is another critical factor that can be enhanced by using graphene. The chemical stability of graphene ensures the sensor maintains its performance over time, even under harsh conditions [30]. Additionally, the rapid electron transfer properties of graphene contribute to faster response times, allowing for real-time monitoring of molecular interactions [31]. This rapid response is essential for applications requiring immediate results, such as point-of-care diagnostics and real-time environmental monitoring.

Numerous studies have demonstrated the benefits of incorporating graphene into SPR biosensors. For instance, graphene-based SPR biosensors have been developed for the detection of various biomolecules, including proteins [32], DNA [33], and SARS-CoV-2 [34]. These sensors have shown improved sensitivity and lower detection limits than traditional SPR biosensors without graphene. The versatility of graphene also allows for the design of multiplexed biosensors, capable of detecting multiple analytes simultaneously, further expanding their applicability.

Despite significant progress in the field, there are still areas that require further investigation, particularly regarding the comprehensive evaluation of performance metrics such as sensitivity, specificity, and stability in SPR biosensors. This review aims to provide a clearer understanding of the impact of graphene on these performance metrics, ultimately guiding future research and development efforts. We point out that there is an extensive body of knowledge on graphene-based SPR biosensors [35,36,37,38,39,40]. However, we aim to consolidate recent information with a concentrated focus on these critical performance metrics. This review seeks to address and close some of the unexplored aspects and gaps in the current literature by synthesizing recent advancements and evaluating how graphene enhances these key parameters. This focused approach will help identify areas needing further exploration, thereby advancing the practical applications and efficacy of SPR biosensors enhanced with graphene.

## 2. Methodology

As noted, the integration of graphene in SPR biosensors represents a significant technological advancement with the potential to revolutionize biosensing applications. However, the field is characterized by a high volume of research with varying methodologies and quality. Therefore, a systematic review is urgently needed to consolidate this diverse body of evidence, identify the most promising approaches, and highlight areas where further research is necessary.

### 2.1. The Review Goal

**Figure 2:**
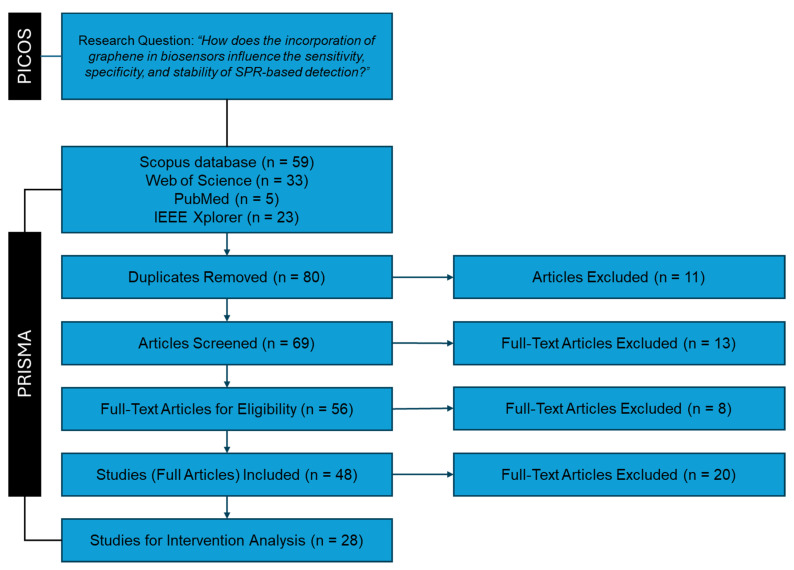
Flowchart of the systematic review process carried out in this work.

The goal of this systematic review is to analyze and synthesize the current state of research on the role of graphene in SPR-based biosensors. Despite the substantial number of narrative reviews available on this topic [36,40], a systematic review is urgently needed to provide a more structured, transparent, and replicable assessment of the existing literature. In this context, the PICOS framework (Population, Intervention, Comparison, Outcomes, and Study Design) [41] is essential for structuring the research question to guide the systematic review process. By clearly defining these components, the PICOS approach ensures that the review addresses specific and relevant aspects of the research topic, thereby improving the focus and relevance of the findings. For this systematic review, Table 1 shows the components of the research question:

Additionally, the PRISMA (Preferred Reporting Items for Systematic Reviews and Meta-Analyses) [42] guidelines provide a standardized approach for reporting systematic reviews and meta-analyses. These guidelines enhance the transparency and completeness of the review by ensuring that all relevant aspects of the review process are thoroughly documented and reported. This includes the search strategy, selection criteria, data extraction, risk of bias assessment, and synthesis of results. This combined approach (PRISMA plus PICOS) ensures that our systematic review, illustrated in Figure 2, encompasses the latest advancements and trends in this field, providing a comprehensive and up-to-date synthesis of the evidence.

### 2.2. Identification Stage

**Figure 3:**
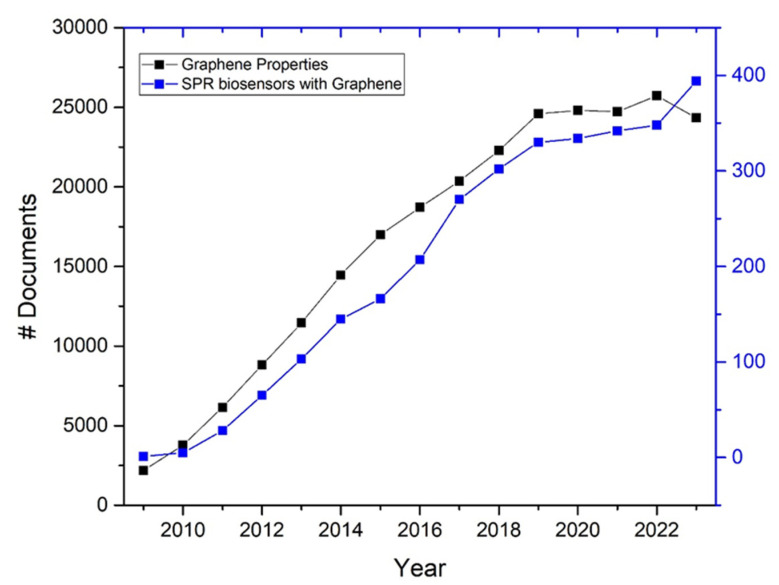
The number of documents identified by searching: “graphene properties and applications” (black) and “integration of graphene in SPR sensors” (blue). Data were obtained from the Scopus database.

The chosen timeframe of 2010 to 2023 for this systematic review is crucial for the following reasons:

- The field of graphene research, particularly its application in biosensors, has seen significant advancements and a surge in scientific publications over the past decade. The unique properties of graphene were more thoroughly understood and explored in this period, leading to numerous innovative applications in biosensing technologies (see Figure 3, black line, data obtained from the Scopus database).
- Starting in 2010, there was a noticeable increase in the number of studies focusing on the integration of graphene in SPR sensors. This period captures the maturation of graphene technology from basic research to more applied studies, including experimental and comparative studies that directly assess the performance improvements brought by graphene (see Figure 3, blue line, data obtained from the Scopus database).

The selection of Scopus, Web of Sciences, PubMed, and IEEE Xplore as the primary databases for this systematic review is strategic and justified by their comprehensive coverage, relevance, and reputation in the scientific community. These databases cover a wide range of scientific disciplines. It provides extensive coverage of journals and conference proceedings, making it an essential resource for a systematic review [43,44,45,46]. By selecting these four databases, the systematic review ensures comprehensive coverage of the relevant literature across multiple disciplines, including materials science, engineering, biomedical research, and applied physics.

Finally, in the identification stage of this systematic review, a precise and comprehensive query strategy was employed to retrieve relevant studies from the selected databases (see Table 2). The query was designed to encompass key terms related to the research topic, ensuring the inclusion of studies that investigate the role of graphene in SPR-based biosensors. The query terms were carefully chosen to capture a wide range of studies while maintaining specificity to the topic.

### 2.3. Screening Stage

We initially identified 120 articles: 59 from Scopus, 33 from Web of Science, 23 from IEEE Xplore, and 5 from PubMed. After removing duplicates, 80 unique articles remained for further screening based on their titles and abstracts. The screening criteria were as follows:

- Inclusion of review articles, full research articles, and proceedings papers.
- Focus on articles specifically addressing graphene.
- Relevance to SPR-based biosensors.
- Inclusion of articles irrespective of language.

During the screening process, 11 articles were excluded, resulting in 69 articles advancing to the next stage. The reasons for exclusion were as follows:

- Eight articles did not focus on or include graphene-related materials.
- One article did not pertain to biosensor technologies.
- Two articles were not available.

### 2.4. Eligibility Stage

During the eligibility phase, the articles were randomly assigned to the authors for a thorough full-text analysis. The eligibility criteria were as follows:

- The full text of the article is available in any language.
- The article focuses on the use of graphene in biosensors.
- The article centers on SPR or related technologies.
- The article specifically discusses the application of graphene in SPR-based biosensors.

At this stage, 13 articles were deemed ineligible, leaving 56 articles suitable for inclusion and data extraction. The reasons for exclusion were as follows:

- Nine articles did not focus on biosensors utilizing graphene.
- Four articles were narrative review papers discussing the general topic of sensors using low-dimensional materials.

### 2.5. Included Stage

To finish, the eligible articles were thoroughly processed to extract all interventions that impact the performance of SPR biosensors. During this stage, 56 articles experienced a comprehensive analysis, with each work evaluated based on the following key metrics:

- Sensitivity.
- Specificity.
- Stability.

At this stage, 8 articles were deemed ineligible, resulting in 48 articles being considered suitable for further extraction and analysis. The reasons for exclusion were as follows:

- Eight articles did not report conclusive metrics. For example, some articles were excluded due to the lack of rigorous experimental methodology to calculate the refractive indices. From the theoretical part, in some studies, models assumed at the same time ideal conditions such as perfect immobilization of biomolecules, absence of non-specific binding, and uniform distribution of the analyte in the sample.

Additionally, out of these 48 articles, 20 were further excluded because they did not pertain exclusively to graphene-based SPR technology. For instance, they were mainly related to surface acoustic wave sensors [47], based optical waveguide sensors [48], and electrochemical sensors [49], among others. Consequently, 28 articles were selected for an in-depth study to contextualize and analyze the role of graphene in enhancing the performance of SPR-based biosensors.

## 3. Results

### 3.1. Summary of Search Results

Initially, a total of 120 articles were identified across the selected databases. After removing duplicates, 80 articles remained for further screening. Following the screening process, 69 articles were selected, and after the eligibility stage, 56 articles were deemed suitable. Ultimately, 24 articles were included in the final data extraction. These articles were analyzed to extract parameters and interventions to contextualize the role of graphene in SPR-based biosensors and its impact on various performance metrics.

The interventions identified/observed in the selected articles were categorized into several key aspects:

- Type of Intervention describes the specific approach or modification applied.
- Type of Biosensor specifies the biosensor types used.
- The Graphene Integration Method details the methods used for incorporating graphene into the biosensors.
- Surface Plasmon Resonance Configuration describes the SPR configurations employed, such as angular, wavelength, or intensity modulation.
- Comparison Material provides a list of the conventional materials used for comparisons, such as gold or silver.

The impact of the interventions on key metrics was observed and categorized:

- Changes in the sensitivity of the biosensors due to graphene integration. Sensitivity refers to the ability to detect small changes in analyte concentration by the variation of refractive indices after biomolecule adsorption. Factors influencing sensitivity include the sensor surface material, surface functionalization quality, and optical setup design.
- Modifications in the specificity of the biosensors when graphene is used. Specificity is the ability to selectively detect the target analyte amidst other non-target molecules. High specificity minimizes false positives and ensures accurate detection of the desired analyte. This is achieved by functionalizing the sensor surface with specific biomolecules like antibodies, aptamers, or DNA that have a high affinity for the target molecule.
- Observed improvements in the stability of the biosensors. Stability refers to maintaining consistent performance over time and under various conditions. High stability ensures the reliability of the sensor over extended periods and different environments. Factors influencing stability include the robustness of materials, surface chemistry, and the ability to withstand environmental changes such as temperature and pH variations.

This thorough extraction and categorization yield a comprehensive understanding of the impact of graphene on SPR-based biosensors, providing insights into the most effective methods and identifying areas for future research.

### 3.2. Summary of Interventions

The comprehensive data extraction is detailed in Table 3, Table A1, Table A2 and Table A3. Specifically, Table 3 provides a summary of the analysis conducted on all articles that progressed to the Inclusion stage.

Table A1 shows a diverse array of SPR biosensors incorporating graphene, utilizing various types and configurations to enhance performance. Most studies focused on traditional SPR sensors with graphene integration methods like deposition on metallic layers (silver, gold) and multilayer structures. Some explored fiber optic SPR sensors, integrating 2D materials such as graphene oxide and molybdenum disulfide for enhanced sensitivity. Additionally, localized surface plasmon resonance (LSPR) sensors leveraged the unique properties of graphene to improve detection capabilities, illustrating its versatility and potential in advancing sensor technology.

Common graphene integration methods included direct deposition of monolayers or thin films on metallic surfaces, advanced techniques like electron beam lithography and nano-sphere lithography for precise placement, transfer printing for coating substrates, and sandwiching graphene between layers in multilayer structures. Innovative methods also involved modeling graphene as surface impedances and using graphene ribbons or elliptic-circular nanodisk resonators, highlighting the adaptability of the material in enhancing SPR biosensor performance through structural and functional enhancements.

The SPR configurations varied widely to optimize performance. The most common setup was the Kretschmann configuration, involving light coupling through a prism to excite surface plasmons on a graphene-coated metal film. Variations included different prism types (e.g., BK7, fused silica), light sources (e.g., helio-neon lasers, TM-polarized plane waves), and additional layers like dielectric gratings or adhesion layers. Some studies used specialized configurations such as fiber optic SPR, long-range SPR (LRSPR), and Surface Plasmon Coupled Emission (SPCE) setups.

### 3.3. Summary of Specific Interventions

Table 3 and Table A2 highlight the diverse specific interventions involving graphene in SPR biosensors aimed at enhancing performance and sensitivity. Specifically, these interventions include incorporating graphene monolayers on metallic layers, using advanced configurations such as periodic subwavelength gratings and multilayer structures, and employing gate-controlled graphene layers for specific applications like glucose sensing. Some studies focused on modeling molecular adsorption effects on the electro-optical properties of graphene, while others combined graphene with materials like molybdenum disulfide and titanium for increased chemical stability. Unique approaches included developing tunable triple-band sensors and using graphene ribbon arrays for infrared detection, demonstrating the innovative use of graphene to address various biosensing challenges.

The target analytes for these graphene-enhanced SPR biosensors span a wide range of biological and chemical substances, reflecting their versatility. Common targets include biomolecular interactions such as cDNA–ssDNA and biotin–streptavidin, which are crucial for genetic and protein analysis. Other specific biomolecules targeted include glucose in blood samples and DNA molecules, underscoring the relevance of these sensors in medical diagnostics. Complex biological entities like Pseudomonas bacteria, hepatitis B virus DNA templates, and COVID-19 virus spike receptor-binding domains are also targeted. Additionally, the sensors aim to detect refractive index changes in gases, liquids, mixed solutions, and substances like H_2_O and D_2_O samples, highlighting their potential for diverse applications in healthcare, environmental monitoring, and biochemical research.

The comparison materials used in these studies serve as benchmarks to evaluate the performance enhancements provided by graphene. Traditional metals like gold and silver are the most frequently used due to their well-established plasmonic properties. Some studies compared graphene-based sensors with bimetallic configurations, such as silver–gold combinations, to assess enhancements in-field performance and sensitivity. Additionally, conventional SPR sensors without graphene, as well as those using other metal thin films like copper, aluminum, and platinum, were used for comparison. Specific studies also employed materials like indium tin oxide (ITO) and transition metal dichalcogenides (TMDCs) such as molybdenum disulfide and tungsten disulfide. The use of these diverse comparison materials emphasizes the comprehensive evaluation of the advantages of graphene in enhancing SPR biosensors, particularly in terms of sensitivity, specificity, and stability across various applications.

### 3.4. Summary of Performance Metrics

Table 3 and Table A3 detail the enhancements in sensitivity, specificity, and stability achieved through various specific interventions involving graphene in SPR biosensors. Sensitivity improvements varied significantly across studies, with some reporting qualitative improvements and others highlighting substantial quantitative gains. These improvements were often linked to the incorporation of graphene layers, with several studies noting enhanced performance as the number of graphene layers increased. Additionally, innovative configurations such as periodic subwavelength gratings, multilayer structures, and specialized sensor designs consistently demonstrated superior sensitivity, underscoring the remarkable impact of graphene on enhancing SPR biosensor performance across diverse applications and configurations.

Specificity improvements were also notable, with many studies reporting qualitative enhancements due to the high adsorption efficiency of graphene and strong interaction with biomolecules. This increased adsorption of target molecules on the graphene surface led to better discrimination between target and non-target substances. The combination of graphene with other materials, like molybdenum disulfide and titanium, further improved specificity. The ability of graphene to form strong π-stacking interactions and its high surface area contributed to improved biomolecule adhesion, enhancing the specificity of the sensor. While precise quantitative improvements were not always provided, the consensus across studies was that graphene significantly enhances the specificity of SPR biosensors, making them more effective in detecting and differentiating target analytes in complex samples.

Additionally, the integration of graphene significantly bolstered the stability of SPR biosensors. The chemical stability of graphene and resistance to oxidation played a crucial role in enhancing overall sensor stability. When layered over metals like silver and gold, the protective properties of graphene prevented oxidation and corrosion, maintaining sensor performance over time. Also, the strong adsorption properties of graphene and its high surface area contributed to more stable biomolecule interactions, leading to consistent and reliable sensor readings. Combining graphene with other materials, such as titanium and molybdenum disulfide, further improved the chemical and thermal stability of the biosensors. In general, these factors made graphene-enhanced SPR biosensors more robust for prolonged and repeated use in various applications.

### 3.5. Data Analysis

Systematic reviews are essential in research due to their ability to minimize bias and ensure comprehensive coverage. They follow a rigorous, predefined methodology that includes an exhaustive literature search and a detailed appraisal of study quality [78]. This approach reduces the risk of bias in selecting and interpreting studies, making the findings more reliable and reproducible [79]. By including a wide range of studies, systematic reviews can identify trends, patterns, and gaps in the research. The quantitative and qualitative synthesis offered by systematic reviews enhances the power and precision of the findings [80].

On the other hand, the PICOS approach, originally developed for healthcare research, is highly beneficial for systematic reviews in natural sciences and engineering such as physics, chemistry, and material science for key reasons. It provides a structured and reproducible framework, ensuring consistency and transparency in the review process [81]. By clearly defining criteria such as Population, Intervention, Comparison, Outcomes, and Study design, PICOS enhances comparability and synthesis of findings across different studies. It also aids in identifying gaps in the literature and areas that require further research, guiding future experimental efforts. Adapting PICOS to natural sciences or engineering allows researchers to leverage its strengths, conducting high-quality systematic reviews that advance knowledge in different fields.

**Figure 4:**
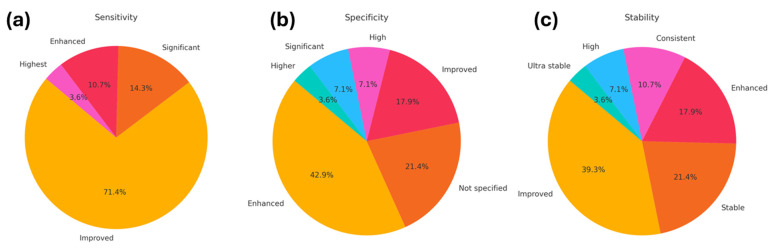
Distribution of qualitative evaluations for performance metrics in graphene-based SPR biosensors. (**a**) Sensitivity improvements, (**b**) specificity enhancements, and (**c**) stability advancements. Each panel represents the proportion of studies reporting different levels of performance enhancement: improved, enhanced, significant, highest, high, higher, ultra stable, and not specified.

With this in mind, we qualitatively analyze the findings observed in the 28 works considered in the Intervention, Table 3. In Figure 4a, a substantial majority of the studies (71.4%) reported an improvement in sensitivity when graphene was integrated into SPR biosensors. A notable proportion (14.3%) reported significant improvements, while smaller percentages noted enhanced (10.7%) and highest (3.6%) sensitivity levels. These findings highlight the crucial role of graphene in boosting sensor sensitivity, making it a valuable material for SPR biosensing applications.

In terms of specificity (Figure 4b), 42.9% of the studies observed enhanced specificity due to graphene integration, highlighting its ability to improve the discrimination between different analytes. Improved specificity was noted in 21.4% of the studies, while a significant improvement was reported in 7.1%. Other categories such as high and higher specificity were noted less frequently (17.9% and 3.6%, respectively), indicating that while graphene generally enhances specificity, the degree of improvement can vary depending on the sensor design and application.

Stability improvements (Figure 4c) were also prominent, with 39.3% of studies reporting improved stability, and 21.4% noting stable performance. Enhanced stability was observed in 17.9% of the studies, with a smaller portion (10.7%) achieving consistent stability. Ultra-stable performance was noted in 7.1%, indicating that graphene not only enhances sensor performance under normal conditions but also provides robustness against environmental variations.

### 3.6. Generated Data

**Figure 5:**
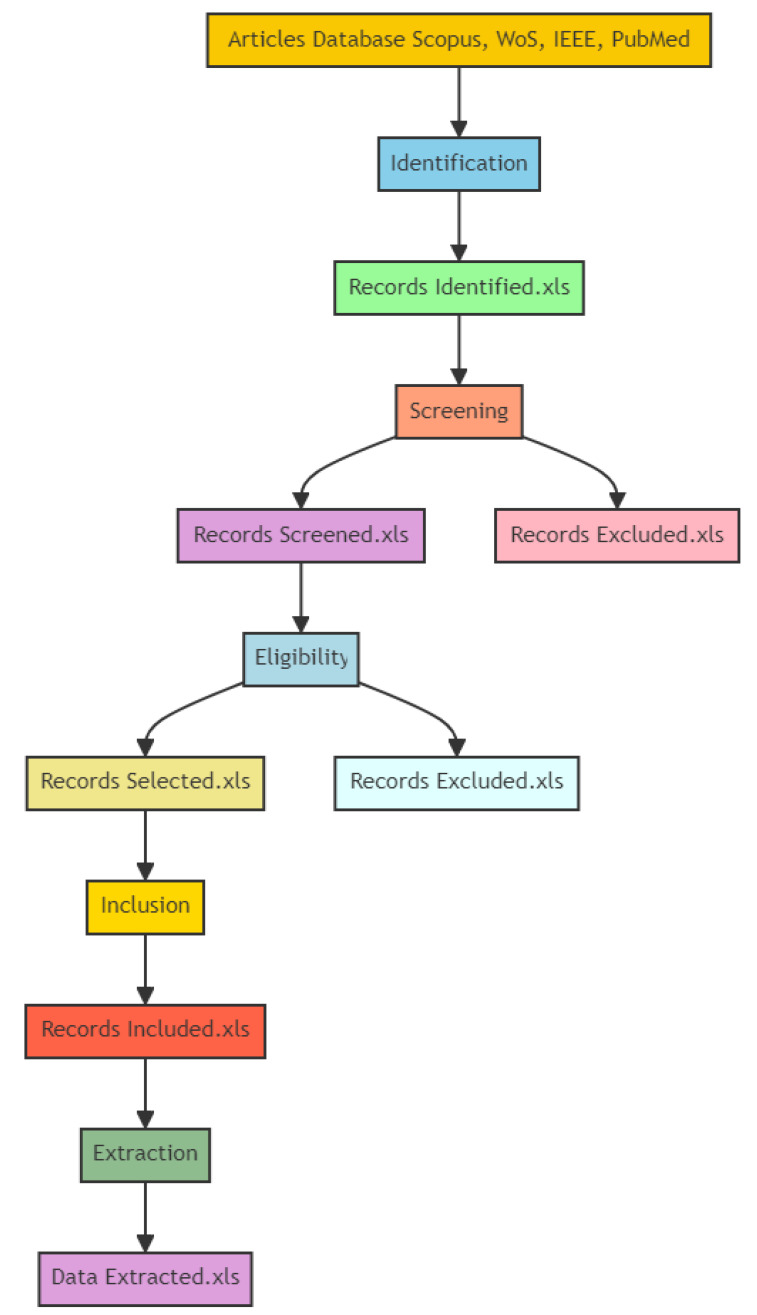
Generated data on the current systematic review process. This diagram illustrates the ordered process of selecting (saving) research articles from various databases such as Scopus, WoS, IEEE, and PubMed. The folder includes the included/excluded records at the different stages.

To warrant clarity and support future research endeavors, we have meticulously recorded each phase of our data generation process within this systematic review. The comprehensive documentation is depicted in Figure 5, outlining the entire review process from the initial article identification to the final data extraction. The flowchart details the following stages:

- Identification;
- Screening;
- Eligibility;
- Inclusion;
- Extraction.

Each stage involves tasks such as identifying records, assessing relevance, selecting eligible studies, and excluding those that do not meet the criteria. This thorough approach ensures a comprehensive and systematic review process.

## 4. Discussion

### 4.1. Enhancements in SPR Biosensors through Graphene Integration

**Figure 6:**
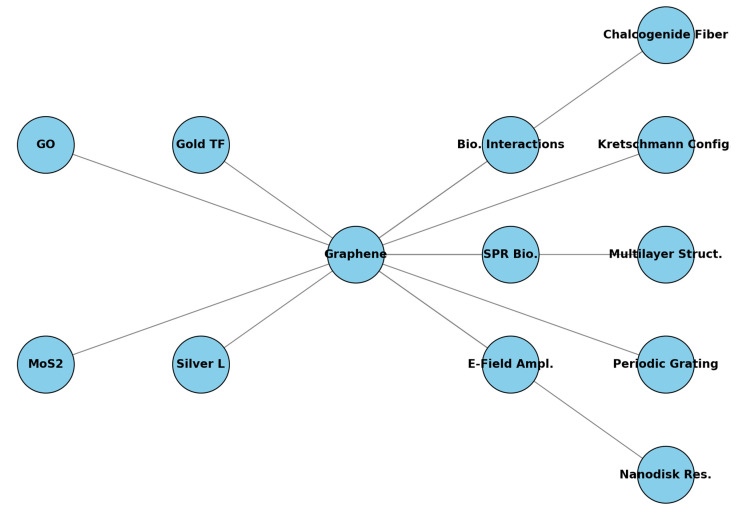
Integration of graphene into SPR biosensors. This figure illustrates the key components and configurations involved in integrating graphene into SPR biosensors. The nodes and their abbreviations are as follows: graphene (Graphene), SPR biosensors (SPR Bio.), gold thin film (Gold TF), silver layer (Silver L), biomolecular interactions (Bio. Interactions), electric field amplification (E-Field Ampl.), Kretschmann configuration (Kretschmann Config.), periodic dielectric subwavelength grating (Periodic Grating), graphene oxide (GO), molybdenum disulfide (MoS2), high-index chalcogenide core fiber (Chalcogenide Fiber), multilayer structure (Multilayer Struct.), and elliptic-circular nanodisk resonators (Nanodisk Res.). The edges depict the relationships and interactions between these components, emphasizing the enhanced performance and capabilities of SPR biosensors through the incorporation of graphene.

The incorporation of graphene into SPR biosensors represents a significant advancement in the field, offering notable improvements in sensitivity, specificity, and stability (Figure 4). Now, Figure 6 aims to convey the multifaceted role of graphene in enhancing the performance of SPR biosensors. Indeed, this discussion emphasizes the critical advancements and improvements graphene brings to SPR biosensors.

One of the most interesting studies in this area is by Islam et al. [51], who demonstrated that a thin graphene layer deposited on a gold thin film, combined with a periodic dielectric subwavelength grating, could significantly amplify the electric field at the sensor surface. This innovative setup, based on the Kretschmann configuration (Figure 1), showed substantial improvements in the detection limits of the biosensor. The periodic grating facilitated efficient light coupling to the plasmonic mode, while the graphene layer enhanced biomolecular interactions due to its high surface area and biocompatibility. This combination resulted in a notable increase in sensitivity and specificity, setting a benchmark for future developments in SPR biosensor technology.

Recently, researchers explored the deposition of a graphene monolayer atop a silver layer, discovering a notable enhancement in the sensitivity of SPR sensors due to the integration of graphene [50]. Utilizing a setup with a helio-neon laser and a BK7 prism, it was found that the exceptional electrical conductivity of graphene significantly amplifies the interaction between the plasmonic field and the analyte, which is crucial for detecting minute analyte concentrations. Expanding on this idea, further research investigated the use of graphene-related materials, such as graphene oxide and molybdenum disulfide, layered on silicon over a silver substrate [52]. The results highlight the ability of these materials to tune the optical properties of the sensor, thereby enhancing its performance. The high-index chalcogenide core fiber configuration, incorporating these 2D materials, demonstrates that graphene and its counterparts provide excellent surfaces for biomolecule binding, essential for enhancing the specificity and stability of the sensor. Theoretical insights into the interaction between sensing molecules and a graphene layer offered a deeper understanding of the changes in the electronic band structure and refractive index, directly influencing the SPR response [53]. These theoretical outcomes explain the fundamental mechanisms by which graphene enhances SPR biosensors, complementing experimental findings with a solid theoretical foundation.

In an innovative approach, an electro-optical SPR biosensor was developed using graphene within a Kretschmann–Raether configuration [54]. The results demonstrate that incorporating graphene significantly boosts sensitivity to various biomolecules. The graphene layer provided a stable and high-affinity surface for analyte binding, essential for achieving high specificity and stability in biosensing applications. Further investigations into a multilayer structure, where graphene layers were modeled as surface impedances with conductivity described by the Kubo formula, revealed that using a THz source to excite this multilayer structure enhances the electromagnetic response of the biosensor [55]. This enhancement led to higher sensitivity and better signal-to-noise ratios. In addition, a novel configuration involving periodically arranged graphene elliptic-circular nanodisk resonators on a dielectric substrate was explored [56]. Simulations using the finite difference time domain (FDTD) method showed that this design could significantly enhance the local electric field. The unique structure of the nanodisks allowed for better control over the plasmonic properties, resulting in improved specificity and stability of the SPR biosensor.

### 4.2. Target Analytes and Comparative Materials

**Figure 7:**
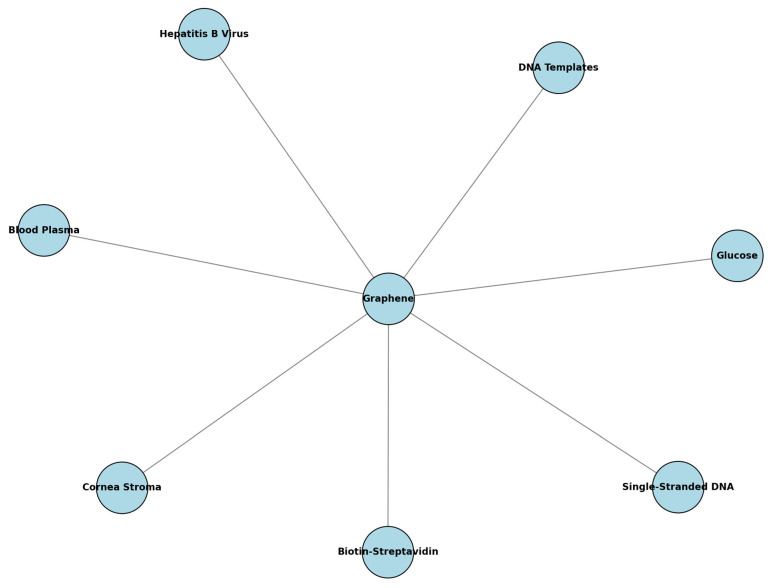
Summary of target analytes studied with graphene-enhanced SPR biosensors.

The integration of graphene into SPR biosensors has led to significant advancements in the detection of various target analytes. Figure 7 illustrates the most preferred target analytes to be detected by using graphene-enhanced SPR biosensors. The central role of graphene in improving the detection capabilities of these biosensors is highlighted. This visualization emphasizes the significant advancements made possible by graphene in the field of SPR biosensors. 

Recent advancements in SPR biosensors have confirmed the utility of integrating graphene to enhance performance and chemical stability. One approach utilized a graphene–molybdenum-enhanced SPR biosensor, incorporating a silver metallic layer and a titanium adhesion layer, to target a range of biological analytes including glucose, corneal stroma, blood plasma, and DNA templates of the hepatitis B virus [57]. This configuration was specifically designed to improve sensitivity and stability. Another study focused on using a graphene sheet coated above a gold thin film for detecting biomolecules with carbon-based ring structures, such as single-stranded DNA [58]. This setup demonstrated superior sensitivity compared to traditional gold thin films, leveraging the high surface area of graphene to enhance interactions with target analytes, thereby improving detection capabilities.

On the other hand, one study involved incorporating a graphene sheet on top of a gold thin film in a localized surface plasmon resonance (LSPR) biosensor to examine the biomolecular interactions of biotin–streptavidin, showcasing higher sensitivity and specificity compared to traditional materials like gold, silver, copper, and aluminum thin films [59]. Further research reinforced these findings by adding a graphene sheet layer on a gold thin film in a variable incidence angle LSPR biosensor, which improved the detection of biotin–streptavidin interactions, outperforming traditional configurations [60]. Another study developed a multilayer LSPR biosensor with an additional graphene sheet layer to enhance sensitivity and detection accuracy for streptavidin interactions [61]. A innovative approach introduced a periodic array of dielectric grating on top of a graphene layer, significantly boosting the sensitivity of an LSPR biosensor for monitoring biomolecular interactions [62].

The application of graphene in SPR biosensors is further extended by developing a sensor using a graphene ribbon array on a quartz substrate for infrared wavelength detection [63]. This configuration aimed at detecting refractive index changes in gases and low-refractive-index materials in aqueous environments and demonstrated superior sensitivity and detection capabilities compared to traditional materials such as metallic nanostructures and metal–dielectric configurations used for SPR sensing in visible and near-infrared wavelengths. The graphene-based approach offered enhanced performance in the infrared region, making it particularly valuable for specific applications requiring infrared detection.

### 4.3. Sensitivity

The integration of graphene into SPR biosensors has led to remarkable advancements in sensitivity, demonstrating the transformative potential of this material in biosensing applications. One notable example is the work of Maharana et al. [50], who reported a more than 22% improvement in sensitivity when using a graphene-based SPR sensor compared to a bimetallic silver–gold configuration. This enhancement was attributed to the superior plasmonic properties of graphene, which enable stronger interactions with the analyte and thus higher sensitivity. Further emphasizing the potential of graphene, Rouf et al. [57] demonstrated that a graphene–molybdenum-enhanced SPR biosensor could achieve a sensitivity 2.42 times higher than conventional SPR sensors and 2.35 times higher than graphene-only sensors. This combination of graphene with molybdenum diselenide shows how the integration of multiple materials can further enhance sensor performance. Adding layers of graphene also proves beneficial, as shown by Wu et al. [58], who achieved up to a 25% improvement in sensitivity with ten layers of graphene compared to conventional gold SPR biosensors. The multilayer structure increases the surface area and the number of interaction sites, significantly boosting the sensitivity. Islam et al. [62] reinforced these findings by showing substantial sensitivity enhancements with the introduction of a graphene layer in their LSPR biosensors. This study highlighted that the sensitivity of graphene-integrated LSPR sensors is significantly higher than that of traditional metal-only LSPR sensors.

Expanding the application range, Wu et al. [63] developed an SPR biosensor using a graphene ribbon array on a quartz substrate, which exhibited extraordinary sensitivity improvements. They reported sensitivities of 4720 nm/RIU for gas detection and 5520 nm/RIU for low-refractive-index materials in aqueous environments, surpassing the performance of traditional materials. Maharana et al. [64] also contributed to this growing body of evidence by showing that the imaging sensitivity of their graphene-based sensor was approximately 50% greater than that of conventional 2S_2_G–gold–graphene-based affinity sensors. This improvement features the potential of graphene to work in imaging applications.

Moreover, Verma et al. [69] demonstrated that their SPR biosensor with metamaterial and graphene exhibited significantly higher sensitivity compared to traditional materials. They quantified these improvements as 54.75°/RIU for metamaterial, 41.47°/RIU for graphene, and 40.42°/RIU for conventional SPR at a wavelength of 750 nm. This study highlights how combining graphene with other advanced materials can lead to substantial sensitivity gains. Last of all, An et al. [70] developed a quasi-D-shaped optical fiber plasmonic biosensor, which showed a wavelength sensitivity ranging from 3908 to 10,693 nm/RIU in a dynamic index range from 1.33 to 1.38. This significant sensitivity improvement over traditional materials demonstrates the effectiveness of incorporating graphene into innovative biosensor designs.

### 4.4. Specificity

The integration of graphene into SPR biosensors has led to significant advancements in specificity, essential for accurately identifying and quantifying target analytes in complex samples. One study highlighted that the high adsorption efficiency of graphene greatly enhances the ability of the sensor to distinguish target analytes [51]. Building on this, combining silver with graphene has been shown to improve detection accuracy over traditional metal-only sensors due to precise control over plasmonic properties [54]. Further research indicated that graphene-based sensors offer superior specificity in the THz range compared to traditional sensors. This is due to the electronic properties of graphene, which facilitate stronger interactions with specific analytes [55]. Additionally, increasing graphene layers improves detection accuracy and specificity, making it easier to differentiate between analytes [71]. A graphene–silicon dioxide/silicon structure has also been shown to enhance specificity through strong coupling conditions between incident light and the graphene structure, leading to more precise detection [73]. Studies consistently report high detection accuracy and strong performance metrics in graphene-based SPR biosensors, highlighting their capability for precise and reliable detection [76,77].

### 4.5. Stability

Stability is crucial for SPR biosensors, ensuring consistent and reliable performance over time and under varying environmental conditions. One study revealed that incorporating graphene into SPR biosensors prevents the oxidation of the silicon layer, maintaining sensor performance over extended periods [52]. Oxidation can degrade sensitivity and accuracy, making the protective role of graphene critical. Another investigation highlighted the high chemical and thermal stability of graphene, bolstering overall sensor durability [53]. Moreover, the chemical stability of graphene and its biocompatibility are essential for preserving biosensor functionality in complex biological environments [56].

Several studies demonstrated notable improvements in stability with the introduction of graphene layers. The strong adsorption of biomolecules on graphene compared to gold ensures more consistent sensor performance. Additionally, graphene acts as a passivating layer, preventing oxidation and corrosion of the gold layer, thus maintaining long-term stability [59,60,65]. Further evidence showed the ability of graphene to prevent oxidation, crucial for extending the operational lifespan of sensors [66]. Another study confirmed that a graphene layer effectively protects against the oxidation of the silver layer in SPR biosensors, ensuring reliability even in oxidative environments [67].

Graphene-based sensors also exhibit resilience under varying environmental conditions. For example, one study found that detection errors remained within acceptable limits despite temperature fluctuations, demonstrating robustness [74]. Additionally, graphene-based sensors maintained high stability and consistent performance, capable of detecting changes in relative permittivity and thickness with high resolution, surpassing the stability of previously reported designs [75]. These findings highlight the transformative role of graphene in enhancing the stability and reliability of SPR biosensors, making them suitable for a wide array of application

## 5. Limitations of the Current Systematic Review

While this systematic review provides a comprehensive analysis of the role of graphene-based SPR biosensors, some limitations should be recognized. These limitations are presented with the intent of providing a balanced perspective without undermining the significance of the findings.

- One of the primary limitations of this review is the relatively limited number of studies focused on the specificity and response time of graphene-enhanced SPR biosensors. While sensitivity and stability metrics were well-represented and thoroughly analyzed, the specificity metrics were based on fewer studies. This limited scope could impact the generalizability of the findings related to these specific performance aspects.
- The studies included in this review employed a wide range of experimental conditions, including different types of graphene, biosensor configurations, target analytes, and detection methods. This variability can make it challenging to directly compare results across studies and may introduce inconsistencies in the reported performance metrics. Although efforts were made to categorize and analyze the studies systematically, the inherent differences in experimental setups should be considered when interpreting the results.
- There is a possibility of publication bias, where studies reporting significant improvements in biosensor performance with graphene integration are more likely to be published than those reporting minimal or no improvements. This bias could skew the overall conclusions of the review, presenting an overly optimistic view of the impact of graphene on SPR biosensors.
- Many of the studies included in this review were conducted under controlled laboratory conditions. There is limited information on the performance and reliability of graphene-enhanced SPR biosensors in realistic applications. Future research should emphasize the testing and validation of these biosensors in practical settings to better understand their applicability and robustness in real-life scenarios.

## 6. Conclusions

This systematic review analyzed the role of graphene in enhancing the performance of SPR-based biosensors. The review covered various aspects, including sensitivity, specificity, and stability across a selection of studies published between 2010 and 2023. By systematically reviewing the literature, we aimed to provide a detailed understanding of how graphene integration influences these critical performance metrics. In particular, the integration of graphene into SPR biosensors represents a significant advancement, offering notable improvements in sensitivity, specificity, and stability. The studies reviewed consistently demonstrated that graphene enhances the sensitivity of SPR biosensors by increasing the surface area for biomolecular interactions and amplifying the electric field at the sensor surface. This results in the ability to detect smaller changes in analyte concentrations, making these sensors more effective for various applications.

The role of graphene in improving specificity is equally important. By providing a more selective detection of target analytes amidst non-target molecules, graphene-functionalized surfaces minimize false positives, thereby ensuring more accurate and reliable detection. This is particularly beneficial in complex biological samples where high specificity is crucial for accurate diagnostics. Stability is another critical metric where graphene has shown significant improvements. The chemical stability of graphene, along with its oxidation resistance, contributes to maintaining consistent sensor performance over extended periods and under various environmental conditions. This makes graphene-enhanced SPR biosensors more robust and reliable for long-term use in diverse applications, ranging from medical diagnostics to environmental monitoring.

Despite these advancements, the review also identified areas needing further exploration. Future research should focus on standardizing experimental conditions to improve comparability across studies and minimize inconsistencies. Additionally, more work is needed to validate the performance of graphene-enhanced SPR biosensors in practical, real-world settings to better understand their applicability and robustness in real-life scenarios.

## Figures and Tables

**Table 1:** PICOS framework was used to structure the research question.

P	Population	Biosensors using surface plasmon resonance (SPR) technology.
I	Intervention	Incorporation of graphene into SPR biosensors.
C	Comparison	SPR biosensors without graphene or using alternative materials.
O	Outcomes	Improved sensitivity, specificity, and stability of SPR-based detection.
S	Study Design	Evaluation of recent research articles and studies on graphene-enhanced SPR biosensors.

**Table 2:** Query type and the corresponding results. Year restriction applied from 2010 to 2023.

Database	Query	Results
Scopus Web of Sciences PubMed IEEE Xplore	(“Surface plasmon resonance”) AND (“Graphene biosensor” OR “Graphene-based biosensor” OR “Graphene sensor” OR “Graphene-based sensor”)	59 33 5 23

**Table 3:** Interventions and Performance metrics.

Reference	Specific Intervention	Target	Sensitivity Improvement	Specificity Improvement	Stability
Maharana et al. [50]	Incorporation of a monolayer of graphene on silver to enhance field and performance	Detection and identification of different biomolecules of carbon-based ring structure	Improved (22%)	Unspecified	Ultra stable
Islam et al. [51]	Incorporation of a periodic array of subwavelength grating on top of a layer of graphene sheet in the multilayer SPR biosensor	Biomolecular interactions of cDNA-ssDNA	Improved	Not specified	Enhanced
Nayak et al. [52]	Incorporation of graphene, graphene oxide, and molybdenum disulfide as sensing layers in an SPR-based biosensor.	Not applicable	Improved (202.2 nm/RIU)	Not specified	Enhanced
Meshingqalam et al. [53]	Modeling the effect of molecular adsorption on the electro-optical properties of graphene-based sensors for SPR detection.	Molecules like graphene–beryllium, graphene–hydrogen, and graphene–calcium, which impact the electro-optical properties of the sensor.	Improved	High	Enhanced
Sharma et al. [54]	Incorporation of a graphene monolayer as a protective and enhancing layer in the SPR sensor.	H_2_O and D_2_O samples.	Enhanced	Enhanced	Stable
Conceicao et al. [55]	Numerical analysis of a graphene-based SPR sensor using the Finite Element Method.	Fluorescent nanoparticles and other compounds in a microfluidic channel	Enhanced	Improved	Enhanced
Liang et al. [56]	Design of a tunable triple-band graphene refractive index sensor with good angle-polarization tolerance	Refractive index changes in the surrounding medium, useful for detecting gases, liquids, or mixed solutions	Improved (11.560 nm/RIU)	Enhanced	Stable
Rouf et al. [57]	The study presents a graphene–molybdenum-enhanced SPR biosensor incorporating a silver metallic layer and a titanium adhesion layer to improve performance and chemical stability.	Biological analytes including glucose, the stroma of the cornea, blood plasma, and DNA templates of the hepatitis B virus	Improved (2.42 times)	Improved	Improved
Wu et al. [58]	Use of graphene sheet coated above a gold thin film	Biomolecules with carbon-based ring structures, such as single-stranded DNA	Improved (25%)	Enhanced	Stable
Islam et al. [59]	Incorporation of a graphene sheet on top of a thin gold film in an LSPR biosensor	Biomolecular interactions of biotin–streptavidin	Improved	Enhanced	Improved
Islam et al. [60]	Introduction of an additional graphene sheet layer on top of a gold thin film in a variable incidence angle LSPR biosensor.	Biotin–streptavidin interaction	Improved (linear)	Improved	Improved
Islam et al. [61]	Introduction of an additional graphene sheet layer on top of a gold layer in a multilayer LSPR biosensor for enhanced sensitivity and detection accuracy.	Streptavidin (biotin–streptavidin interactions)	Enhanced	Improved	Improved
Islam et al. [62]	Introduction of a periodic array of dielectric grating on top of a graphene layer to improve the sensitivity of an LSPR biosensor for monitoring biomolecular interactions of biotin–streptavidin.	Biotin–streptavidin interactions	Significant	Enhanced	Improved
Wu et al. [63]	Development of a SPR biosensor using a graphene ribbon array on a quartz substrate for infrared wavelength detection.	Detection of refractive index changes in gases and low-refractive-index materials in aqueous environments.	Improved (4720 nm/RIU)	Enhanced	Stable
Maharana et al. [64]	Development of a low-index dielectric (Teflon)-mediated SPR sensor based on graphene in a dielectric–metal–dielectric configuration for near-infrared measurements.	Specific biomolecules detectable in the near-infrared spectrum	Improved (50%)	Enhanced	Improved
Islam et al. [65]	Integration of a periodic array of subwavelength grating on top of a layer of graphene sheet to improve sensitivity for DNA hybridization detection.	Biomolecular interactions, specifically focusing on the binding of biotin–streptavidin	Improved (18%)	Enhanced	Improved
Toloue et al. [66]	The study incorporates graphene layers on a conventional copper SPR biosensor to enhance sensitivity. This is based on the high adsorption efficiency of graphene due to π-stacking interaction with carbon-based ring biomolecules like single-stranded DNA.	DNA molecules	Significant	Not specified	Improved
Verma et al. [67]	Use of graphene and an air gap as dielectric layers in the SPR biosensor	Detection of Pseudomonas and Pseudomonas-like bacteria	Improved (2.35 times)	Not specified	Improved
Wu et al. [68]	Incorporation of a graphene layer on the metal surface of a LRSPR biosensor.	Enhancing the sensitivity and detection accuracy of biosensors for chemical examination, medical diagnosis, and biological detection.	Significant (Nearly tenfold)	Significant	Improved
Verma et al. [69]	The study proposes to use graphene/metamaterial film to enhance the adsorption of biomolecules. The film of graphene/metamaterial is coated on a gold film in the conventional SPR biosensor.	Biomolecule detection	Improved (750 nm: 54.75°/RIU)	Significant	Enhanced
An et al. [70]	The study involves the use of chemically stable graphene and indium tin oxide (ITO) layers outside the fiber structure to realize a simple detection mechanism.	Refractive index sensing for biomolecules, water quality analysis, and other analytes.	Improved (1069–3906 nm/RIU)	Enhanced	Improved
Huang et al. [71]	Use of continuous suspended monolayer graphene (MLG) and graphene/insulator stacks (GIS) for SPR-based THz plasmonic sensing	Enhancing detection accuracy and sensitivity for gas sensing applications	Significant (Up to 51.0°/RIU)	Higher	Consistent
Lin et al. [72]	Use of graphene as a defect layer attached to the surface of a one-dimensional photonic crystal (1DPC) to enhance biosensor performance. SPR biosensors rely on the excitation of surface plasmons on a metal layer, and BSW biosensors use a 1DPC to excite Bloch Surface Waves	The target for the biosensor is not explicitly mentioned, but it is designed to enhance sensitivity and detection accuracy, likely for various biomolecules.	Improved (3.5 times)	Not specified	Stable
Farmani et al. [73]	Use of a monolayer of chemical vapor deposition (CVD) graphene as the sensing layer.	High-resolution detection of refractive index changes in environmental monitoring applications, including temperature sensing and photodetectors for atomic force microscopy	Highest	Improved	Stable
Hossain et al. [74]	Gate-controlled graphene SPR glucose sensor	Detection of glucose in blood samples	Improved (21.4%)	Enhanced	Consistent
Behboudi et al. [75]	Use of a graphene-based metasurface for THz sensing	Wide range of biological tissues and chemical compounds.	Improved (1.5 THz/Permittivity)	Enhanced	High
Mostufa et al. [76]	Incorporation of a graphene-based multilayered structure (BK7/tungsten disulfide/gold/barium titanate/graphene) for an SPR biosensor designed for rapid detection of the novel coronavirus (COVID-19).	Virus spike receptor-binding domain (RBD) and interactions with monoclonal antibodies (mAbs).	Improved (230.77°/RIU)	High	Consistent
Ishtiak et al. [77]	Incorporation of graphene for enhanced sensitivity in water salinity detection using SPR.	The concentration of salinity in water	Improved (397.1°/RIU)	Enhanced	High

## Data Availability

Raw Data are available at https://doi.org/10.17605/OSF.IO/HWPQG.

## Appendix A

**Table A1:** Interventions considering the type of biosensor, graphene integration method, and SPR configuration.

Reference	Type of Biosensor	Graphene Integration Method	SPR Configuration
Maharana et al. [50]	SPR	Deposition of a monolayer of graphene on top of the silver layer	The setup consists of a helio–neon laser (632.8 nm), a polarizer, a BK7 prism on a high-precision rotary stage, and a photodetector. The optimized thickness of silver and graphene is deposited on the prism surface.
Islam et al. [51]	SPR	Deposition of a 2 nm thick graphene layer on top of a 50 nm gold thin film, followed by placing a periodic dielectric subwavelength grating on top of the graphene layer	Kretschmann configuration with a fused silica glass prism, gold thin film (50 nm), and a graphene layer (2 nm), over which a periodic dielectric subwavelength grating made of polymethyl methacrylate (PMMA) is placed. The setup uses a TM-polarized plane wave light of fixed wavelength 632.8 nm.
Nayak et al. [52]	Fiber optic sensor based on SPR	Use of 2D materials (graphene, graphene oxide, molybdenum disulfide) on silicon over a silver layer.	High-index chalcogenide core fiber with a silicon overlayer and different 2D materials on top of silver.
Meshingqalam et al. [53]	SPR	The study models the interaction of sensing molecules with a graphene layer to observe changes in its electronic band structure and refractive index, influencing SPR response.	The study involves modeling the refractive index variations and SPR response using a transverse magnetic wave model in a graphene-based sensor configuration.
Sharma et al. [54]	SPR	Graphene-based electro-optical SPR biosensor	Kretschmann–Raether configuration-based SPR sensor with a ZBLAN–silver–graphene-analyte structure.
Conceicao et al. [55]	SPCE	Graphene layers are modeled as surface impedances with conductivity described by the Kubo formula.	Multilayer structure: air/graphene/silicon dioxide/graphene/prism, excited by a THz source.
Liang et al. [56]	SPR	Periodically arranged graphene elliptic-circular nanodisk resonators on a dielectric substrate.	Graphene elliptical and circular nanodisks are periodically arranged on a dielectric substrate, utilizing the finite difference time domain (FDTD) method for simulations.
Rouf et al. [57]	SPR	The graphene layer is integrated using a layered structure where it is sandwiched between the metal silver and the sensing layers, serving as a protective layer and biomolecular recognition element.	The configuration includes a BK7 prism, Ti adhesion layer, silver layer, molybdenum diselenide layer, and graphene layer. The sensor operates with p-polarized monochromatic light, optimized at a 750 nm excitation wavelength.
Wu et al. [58]	SPR	Coating of graphene on gold surface using a transfer printing technique	Kretschmann configuration with a prism coupler
Islam et al. [59]	LSPR	Coating of a graphene layer on top of the gold thin film	Kretschmann configuration with a prism coupler
Islam et al. [60]	LSPR	The graphene sheet is deposited on top of the gold layer.	Kretschmann configuration with a triangular prism and variable incidence angles, using a gold thin film with a graphene layer on top, operating with a fixed wavelength of 632.8 nm.
Islam et al. [61]	LSPR	The graphene sheet is deposited on top of the gold layer using electron beam lithography and nano-sphere lithography.	Kretschmann configuration with a triangular prism, various incidence angles, and a 632.8 nm laser beam.
Islam et al. [62]	LSPR	The graphene layer is incorporated on top of a thin gold film, with periodic dielectric grating placed on top.	Kretschmann configuration with a thin gold film, graphene layer, and periodic dielectric grating on top. The setup includes a fused silica glass prism and a TM-polarized plane wave light of 632 nm.
Wu et al. [63]	SPR	Graphene ribbons were fabricated and integrated into the sensor design through a periodic array on a quartz substrate.	The sensor configuration includes a graphene ribbon array on top of a quartz (silicon dioxide) substrate. The SPR effect is measured by detecting spectral shifts of the resonant transmission dip due to changes in the refractive index of the medium above the sensor.
Maharana et al. [64]	SPR	Graphene layers were incorporated on top of a gold film, forming part of a multilayer structure.	The sensor configuration includes a chalcogenide glass prism, a teflon layer, gold film, and a graphene layer. The SPR setup utilizes the Kretschmann configuration, where TM polarized light is launched at the prism and reflectivity is measured.
Islam et al. [65]	SPR	A graphene layer is deposited on top of a gold thin film, followed by a periodic subwavelength dielectric grating.	Kretschmann configuration with a gold thin film, graphene layer, and periodic dielectric grating. The setup includes a fused silica glass prism, and the excitation is achieved using TM-polarized plane wave light at 632.8 nm.
Toloue et al. [66]	SPR	Graphene layers are added on top of the copper thin film as a binding recognition element (BRE).	The setup includes a 50 nm copper film with multiple layers of graphene on one side and SF10 glass on the other. The thickness of each graphene layer is 0.34 nm, and the sensor operates at a fixed wavelength of 633 nm using a helio-neon laser.
Verma et al. [67]	SPR	The graphene layer is coated on a silver layer, which is deposited on a chromium substrate	Kretschmann configuration with a prism (SF10), air gap, chromium layer, silver layer, graphene layer, affinity layer, and sensing medium
Wu et al. [68]	LRSPR	Graphene layer coated on a metal thin film (such as copper, aluminum, or silver) deposited on a chalcogenide glass (2S2G) prism with a cytop thin film.	The setup involves a 2S2G glass prism, cytop thin film, metal thin film, and the graphene layer. The excitation light wavelength used is 633 nm.
Verma et al. [69]	SPR	Graphene is integrated into the gold film, showing stable adsorption of carbon-based biomolecules (e.g., ssDNA).	Kretschmann configuration, where a high refractive index prism is coated with a metal film touching the sensing medium.
An et al. [70]	Quasi-D-Shaped Optical Fiber Plasmonic Biosensor	Graphene is deposited over ITO, which is then coated on the polished plane of the quasi-D-shaped photonic crystal fiber (PCF).	The configuration involves a quasi-D-shaped PCF with a short polishing depth. ITO coated with graphene is deposited on the polished plane. The sensing mechanism is based on the wavelength interrogation sensitivity of the sensor.
Huang et al. [71]	THz sensor. This sensor operates using the SPR phenomenon at terahertz frequencies	Continuous suspended monolayer graphene (MLG) and graphene/insulator stacks (GIS).	Otto configuration with a prism, analyte, substrate, and MLG or GIS layers. The GIS is separated by a buffer layer with a thickness of 20 nm and a refractive index of 1.535, and deposited on a substrate with a refractive index of 1.6. In the suspended MLG configuration, the substrate refractive index was set to 1
Lin et al. [72]	Intensity-sensitive Bloch Surface Wave (BSW) biosensor.	Graphene layers are used as defect layers on the surface of 1DPC, with the number of layers optimized for performance.	Kretschmann configuration with a ZF10 half-cylinder coupling prism, 1DPC composed of titanium dioxide and silicon dioxide layers.
Farmani et al. [73]	SPR	Monolayer CVD graphene was transferred onto silicon dioxide/silicene substrate using poly(methyl methacrylate) (PMMA) as a supporting layer, followed by etching of PMMA with dilute hydrochloric acid.	Otto configuration, an attenuated total reflection (ATR)-SPR setup with incident light system, Otto configuration system, and reflected light detection system.
Hossain et al. [74]	Optical biosensor for glucose detection	Modification of mono-layer graphene Fermi energy (*Ef*) by a gate voltage (*Vg*)	Transfer matrix method (TMM)-based angular interrogation technique, using incident light with a 633-nm wavelength and TM polarization
Behboudi et al. [75]	THz sensor	Perforated stripe with periodic grooves made of graphene, laid over a polyimide substrate	The sensor operates in reflection mode using a photoconductive antenna (PCA) as the THz source in its pulse mode, operating over a frequency range of 0.2–6 THz
Mostufa et al. [76]	SPR	Graphene is integrated as a monolayer in a multilayered structure with other materials including tungsten disulfide and barium titanate.	Kretschmann configuration with a p-polarized light source (helio-neon laser) at a wavelength of 633 nm incident on a BK_7_ prism, detectors (CCD or CMOS) measure output reflection intensity and angle of incidence.
Ishtiak et al. [77]	SPR	Graphene integrated through deposition methods (details on deposition methods for other layers are provided).	Calcium fluoride prism as substrate, zinc oxide layer for adhesion, silver layer, thin Si layer, and graphene layer. Multiple light sources are used for detection.

**Table A2:** Specific intervention by considering the target analyte and material used for comparison.

Reference	Specific Intervention	Target	Comparison Material
Maharana et al. [50]	Incorporation of a monolayer of graphene on silver to enhance field and performance	Detection and identification of different biomolecules of carbon-based ring structure	Bimetallic silver–gold configuration
Islam et al. [51]	Incorporation of a periodic array of subwavelength grating on top of a layer of graphene sheet in the multilayer SPR biosensor	Biomolecular interactions of cDNA–ssDNA	The traditional gold thin film without graphene, a conventional SPR biosensor, and a graphene-based SPR biosensor (G-SPRB)
Nayak et al. [52]	Incorporation of graphene, graphene oxide, and molybdenum disulfide as sensing layers in an SPR-based biosensor.	Not applicable	Silver without any 2D materials.
Meshingqalam et al. [53]	Modeling the effect of molecular adsorption on the electro-optical properties of graphene-based sensors for SPR detection.	Molecules like graphene–beryllium, graphene–hydrogen, and graphene–calcium, which impact the electro-optical properties of the sensor.	Not explicitly mentioned, but the study focuses on the changes in graphene properties due to molecular adsorption.
Sharma et al. [54]	Incorporation of a graphene monolayer as a protective and enhancing layer in the SPR sensor.	H_2_O and D_2_O samples.	Silver layer used alone, and potential use of silver–gold bimetallic layers.
Conceicao et al. [55]	Numerical analysis of a graphene-based SPR sensor using the Finite Element Method.	Fluorescent nanoparticles and other compounds in a microfluidic channel	Traditional SPCE sensor models use gold and operate in the optical spectrum.
Liang et al. [56]	Design of a tunable triple-band graphene refractive index sensor with good angle-polarization tolerance	Refractive index changes in the surrounding medium, useful for detecting gases, liquids, or mixed solutions	Traditional SPR sensors typically use metals like gold and silver.
Rouf et al. [57]	The study presents a graphene–molybdenum-enhanced SPR biosensor incorporating a silver metallic layer and a titanium adhesion layer to improve performance and chemical stability.	Biological analytes including glucose, the stroma of the cornea, blood plasma, and DNA templates of the hepatitis B virus	Traditional materials such as gold and conventional SPR sensors without molybdenum diselenide and graphene.
Wu et al. [58]	Use of graphene sheet coated above a gold thin film	Biomolecules with carbon-based ring structures, such as single-stranded DNA	Gold thin film
Islam et al. [59]	Incorporation of a graphene sheet on top of a gold thin film in an LSPR biosensor	Biomolecular interactions of biotin–streptavidin	Gold, silver, copper, and aluminum thin films
Islam et al. [60]	Introduction of an additional graphene sheet layer on top of a gold thin film in a variable incidence angle LSPR biosensor.	Biotin–streptavidin interaction	Traditional materials such as gold and configurations without the graphene layer.
Islam et al. [61]	Introduction of an additional graphene sheet layer on top of a gold layer in a multilayer LSPR biosensor for enhanced sensitivity and detection accuracy.	Streptavidin (biotin–streptavidin interactions)	Traditional materials such as gold, silver, copper, and aluminum thin films.
Islam et al. [62]	Introduction of a periodic array of dielectric grating on top of a graphene layer to improve the sensitivity of an LSPR biosensor for monitoring biomolecular interactions of biotin–streptavidin.	Biotin–streptavidin interactions	Traditional materials include gold, copper, silver, and aluminum thin films.
Wu et al. [63]	Development of a SPR biosensor using a graphene ribbon array on a quartz substrate for infrared wavelength detection.	Detection of refractive index changes in gases and low-refractive-index materials in aqueous environments.	Traditional materials such as metallic nanostructures and metal–dielectric configurations are used for SPR sensing in visible and near-infrared wavelengths.
Maharana et al. [64]	Development of a low-index dielectric (Teflon)-mediated SPR sensor based on graphene in a dielectric-metal–dielectric configuration for near-infrared measurements.	Specific biomolecules detectable in the near-infrared spectrum	Traditional materials used for comparison include gold and silver
Islam et al. [65]	Integration of a periodic array of subwavelength grating on top of a layer of graphene sheet to improve sensitivity for DNA hybridization detection.	Biomolecular interactions, specifically focusing on the binding of biotin–streptavidin	Gold thin films and other metal–dielectric configurations are used in traditional SPR sensors.
Toloue et al. [66]	The study incorporates graphene layers on a conventional copper SPR biosensor to enhance sensitivity. This is based on the high adsorption efficiency of graphene due to π-stacking interaction with carbon-based ring biomolecules like single-stranded DNA.	DNA molecules	Traditional materials compared in the study include copper alone without the addition of graphene.
Vermaet al. [67]	Use of graphene and an air gap as dielectric layers in the SPR biosensor	Detection of Pseudomonas and Pseudomonas-like bacteria	Silver and gold
Wu et al. [68]	Incorporation of a graphene layer on the metal surface of a LRSPR biosensor.	Enhancing the sensitivity and detection accuracy of biosensors for chemical examination, medical diagnosis, and biological detection.	Traditional materials used for comparison include gold, copper, aluminum, and silver.
Verma et al. [69]	The study proposes to use a graphene/metamaterial film to enhance the adsorption of biomolecules. The film of graphene/metamaterial is coated on a gold film in the conventional SPR biosensor.	Biomolecule detection	The study compares the proposed biosensor with existing two-dimensional nanomaterials such as graphene-based biosensors and conventional SPR biosensors using gold or silver.
An et al. [70]	The study involves the use of chemically stable graphene and indium tin oxide (ITO) layers outside the fiber structure to realize a simple detection mechanism.	Refractive index sensing for biomolecules, water quality analysis, and other analytes.	The study compares the proposed biosensor with traditional D-shaped optical fibers and other conventional SPR-based sensors.
Huang et al. [71]	Use of continuous suspended monolayer graphene (MLG) and graphene/insulator stacks (GIS) for SPR-based THz plasmonic sensing	Enhancing detection accuracy and sensitivity for gas sensing applications	Traditional materials are not explicitly listed, but the study focuses on the advantages of graphene over noble metal plasmons.
Lin et al. [72]	Use of graphene as a defect layer attached to the surface of a one-dimensional photonic crystal (1DPC) to enhance biosensor performance. SPR biosensors rely on the excitation of surface plasmons on a metal layer, and BSW biosensors use a 1DPC to excite Bloch surface waves	The target for the biosensor is not explicitly mentioned, but it is designed to enhance sensitivity and detection accuracy, likely for various biomolecules.	Traditional BSW sensors without graphene, and conventional SPR biosensors.
Farmani et al. [73]	Use of a monolayer of chemical vapor deposition (CVD) graphene as the sensing layer.	High-resolution detection of refractive index changes in environmental monitoring applications, including temperature sensing and photodetectors for atomic force microscopy	Traditional materials such as noble metals (gold and silver) are used in conventional SPR configurations.
Hossain et al. [74]	Gate-controlled graphene SPR glucose sensor	Detection of glucose in blood samples	Gold-coated dielectric materials, Au–chromium nano-laminated SPR sensors
Behboudi et al. [75]	Use of a graphene-based metasurface for THz sensing	Wide range of biological tissues and chemical compounds.	Noble metals such as gold, copper, and platinum
Mostufa et al. [76]	Incorporation of a graphene-based multilayered structure (BK7/tungsten disulfide/gold/barium titanate/graphene) for SPR biosensor designed for rapid detection of the novel coronavirus (COVID-19).	Virus spike receptor-binding domain (RBD) and interactions with monoclonal antibodies (mAbs).	Traditional materials such as gold, platinum diselenide, and transition metal dichalcogenides (TMDCs) like molybdenum disulfide and tungsten disulfide.
Ishtiak et al. [77]	Incorporation of graphene for enhanced sensitivity in water salinity detection using SPR.	The concentration of salinity in water	Silver layer, compared with existing works.

**Table A3:** Performance metrics of graphene-based SPR biosensors.

Reference	Sensitivity Improvement	Specificity Improvement	Stability	Key Results
Maharana et al. [50]	More than 22% higher sensitivity compared to bimetallic silver–gold configuration	The study does not explicitly mention specificity improvement percentages but indicates enhanced biomolecule adhesion due to π-stacking interaction with graphene.	Graphene prevents oxidation of silver, resulting in an ultra-stable biosensor	Incorporation of a graphene monolayer on silver enhances the electric field by over 30% compared to the silver–gold bimetallic configuration. The proposed graphene-based sensor has more than 38% narrower full-width at half-maximum (FWHM) than the bimetallic configuration, indicating better detection accuracy. The graphene monolayer improves biomolecule adhesion, providing better interaction with the sensing layer and leading to higher performance in real-time biomolecular interactions.
Islam et al. [51]	Improved (specific percentage not provided, but sensitivity increases compared to both conventional SPRB and G-SPRB)	Enhanced (qualitative improvement indicated, the specific percentage not provided)	Graphene prevents oxidation of the underlying metal layer, resulting in more stable performance over time	The incorporation of a periodic array of subwavelength grating on a graphene layer significantly enhances the sensitivity of the SPR biosensor. The proposed GG-SPRB (graphene grating-based SPR biosensor) shows a larger shift in the resonance peak angle compared to conventional SPRB and G-SPRB, indicating better sensitivity. The study demonstrates improved field amplification and coupling of surface plasmon polaritons (SPPs) in the dielectric grating, leading to enhanced detection performance. The tunable operation of the GG-SPRB allows for optimized performance by varying design parameters, such as the number of graphene layers and the thickness of the biomolecular layer.
Nayak et al. [52]	Sensitivity increases from 190.47 nm/RIU for a single layer to 196.63 nm/RIU for five layers of graphene. The GO-based sensor showed a sensitivity of 202.2 nm/RIU compared to 189.4 nm/RIU for a graphene-based sensor.	Specificity improvement is not explicitly mentioned, but the study indicates better performance in terms of FOM (Figure of Merit) for graphene oxide and molybdenum disulfide compared to graphene.	Graphene and its derivatives, such as graphene oxide, enhance stability by preventing the oxidation of the silicon layer.	The incorporation of graphene and its derivatives significantly enhances the sensitivity and electric field intensity of SPR-based biosensors. Graphene oxide shows the highest sensitivity (202.2 nm/RIU) and lowest FWHM, resulting in the highest Figure of Merit (FOM). The electric field enhancement for the silver–silicon–graphene oxide system is 4.65 × 10^4^%.
Meshingqalam et al. [53]	Sensitivity is improved due to the high specific surface area and attractive properties of graphene, such as high electron mobility and low electrical noise. Quantitative improvements are not provided but the improvement is noted as significant due to these factors.	Specificity improvement is not explicitly quantified, but the study notes the use of graphene enhances detection capabilities due to its unique electronic properties and high adsorption efficiency for sensing molecules.	The stability of graphene-incorporated biosensors is enhanced due to graphene’s high chemical and thermal stability, as well as its functionalization capability which makes it suitable for high-performance label-free chemical sensing.	The incorporation of graphene significantly enhances the sensitivity and electrical properties of SPR-based biosensors. The adsorption of sensing molecules on graphene’s surface leads to changes in its electrical conductivity, which can be attributed to local carrier concentration changes. Refractive index deviations based on band gap variations are modeled, showing that sensing molecule adsorption results in a non-zero band gap and changes in the refractive index, ultimately affecting SPR responses.
Sharma et al. [54]	Qualitative improvement: Enhanced sensitivity observed in the near-IR region for graphene monolayers. The combined sensitivity factor (CSF) can be maximized for a chemical potential range of 0.7 < µ < 1 eV.	Enhanced detection accuracy compared to traditional metal-only sensors due to the narrow spectral width of silver combined with graphene.	Graphene provides a protective coating atop the silver layer, preventing oxidation and improving chemical stability.	The study demonstrates that the incorporation of graphene in the “ZBLAN fluoride glass–silver–graphene” plasmonic structure significantly enhances the sensing performance. Optimal performance is achieved with a graphene monolayer and a chemical potential between 0.7 and 1 eV. Using multilayer graphene can slightly improve sensitivity, but the combined performance parameter (CSF) decreases with increased layers due to the damping effect.
Conceicao et al. [55]	The study indicates enhanced sensitivity due to the dynamic doping of graphene, although a specific percentage is not provided.	The graphene-based sensor shows improved specificity in the terahertz range compared to traditional SPCE sensors operating in the optical spectrum.	The study discusses the stability of the graphene-incorporated sensor, noting the enhanced stability provided by the dynamic control of graphene’s surface conductivity.	The study demonstrates that the graphene-based SPCE sensor has better performance in terms of sensitivity and specificity in the terahertz range compared to traditional gold-based SPCE sensors in the optical spectrum. The dynamic doping of graphene layers significantly influences the sensor’s response.
Liang et al. [56]	The article does not provide a specific percentage improvement in sensitivity but indicates a sensitivity of up to 11.56 μm/RIU	Enhanced	Good chemical stability, and high biocompatibility.	The study demonstrates that the graphene-based sensor has good angle-polarization tolerance and maintains polarization insensitivity over a wide angular range (0–60°). The sensor shows a large sensing range and high sensitivity, with a sensitivity of up to 11.56 μm/RIU. The sensing characteristics can be actively adjusted by changing the doping level of graphene, which is not possible with conventional SPR sensors. The graphene sensor can monitor tiny variations in the refractive index, making it suitable for detecting gases, liquids, or mixed solutions.
Rouf et al. [57]	Results are 2.42 times higher than the conventional SPR sensor and 2.35 times higher than the graphene-based sensor.	The improved sensitivity indicates better specificity as well.	Improved stability due to the inclusion of a Ti adhesion layer that protects the silver layer from oxidation, and graphene providing a protective layer.	The proposed sensor shows significant sensitivity enhancement with a sensitivity of 215.5°/RIU. The quality factor is slightly compromised, being 86% and 88% of conventional and graphene-based sensors, respectively. The sensor effectively detects biological analytes such as glucose, the stroma of the cornea, blood plasma, and DNA templates of the hepatitis B virus (HBV). The sensitivity improvement is due to the enhanced evanescent fields and increased interaction volume.
Wu et al. [58]	Up to 25% improvement in sensitivity with 10 layers of graphene compared to conventional gold SPR biosensors	The study does not explicitly mention specificity improvement percentages but indicates enhanced adsorption efficiency due to graphene’s interaction with biomolecules.	Improved stability due to stronger and more stable adsorption of biomolecules on graphene	The incorporation of graphene on gold thin films in SPR biosensors leads to a substantial increase in sensitivity, which can be attributed to two main factors: (a) the strong and stable adsorption of biomolecules on graphene, and (b) the modification of SPR curves by the optical properties of graphene. The overall sensitivity is increased by a factor of (1 + 0.025 L) × γ, where γ > 1.
Islam et al. [59]	Improved (specific percentage not provided, but sensitivity increases almost linearly with the number of graphene layers)	Enhanced (qualitative improvement mentioned, but no specific percentage provided)	Improved stability due to the stronger adsorption of biomolecules on graphene compared to gold, resulting in consistent performance	The study demonstrated that the incorporation of a graphene layer on top of a gold thin film in an LSPR biosensor significantly enhanced both sensitivity and adsorption efficiency compared to conventional LSPR biosensors. Sensitivity improvements were influenced by the number of graphene layers and the operating wavelength. The graphene-on-gold LSPR biosensor exhibited better sensitivity at lower operating wavelengths and with a larger number of graphene layers.
Islam et al. [60]	The sensitivity increases with the addition of graphene and is linearly related to the number of graphene layers.	The improved sensitivity indicates better specificity.	Improved stability due to the introduction of the graphene layer, which enhances the adsorption of biomolecules.	The study investigates the enhancement of the sensitivity of a variable incidence angle LSPR biosensor by introducing an additional graphene sheet layer on top of the gold thin film. The sensitivity, indicated by the shift of the plasmon resonance angle, increases with graphene deposited onto the gold layers and is linearly related to the number of graphene layers. The investigation was carried out for different analyte interfaces (air and water), and it was found that the graphene biosensor has better sensitivity for triangular prisms, higher prism angles, and water interfaces.
Islam et al. [61]	Enhanced sensitivity was observed with the introduction of graphene layers, with an overall improvement indicated by a higher shift in the plasmon dip compared to traditional materials.	Enhanced specificity due to the increased adsorption efficiency of graphene.	Improved stability with the inclusion of a graphene layer and an additional layer of silica-doped boron trioxide (sdB_2_O_3_), which compensates for the reduced signal-to-noise ratio.	The study investigates the improvement in sensitivity, adsorption efficiency, and detection accuracy of a multilayer LSPR graphene biosensor. The sensitivity of the conventional LSPR biosensor is enhanced by using the graphene sheet as a biomolecular recognition element on top of the gold thin film. The sensitivity can be further improved by selecting the appropriate operating wavelength, prism configuration, interface with prism, and thickness of target biomolecules. Introducing a layer of sdB_2_O_3_ under the graphene layer significantly improves the signal-to-noise ratio (SNR), though sensitivity is slightly reduced. The proposed design achieves a balance between high sensitivity and high detection accuracy.
Islam et al. [62]	The study shows a significant enhancement in sensitivity with the introduction of a graphene layer, resulting in improved sensitivity compared to traditional metal-only LSPR sensors.	Enhanced specificity due to the stronger adsorption of biomolecules on the graphene layer.	Improved stability due to the incorporation of graphene, which enhances the adsorption efficiency and stability of the biosensor.	The study presents a multilayer design for an LSPR biosensor incorporating a graphene layer and a periodic array of dielectric grating on top. The proposed design significantly improves the sensitivity of the LSPR biosensor for monitoring biomolecular interactions. The sensitivity enhancement is attributed to the creation of local hot spots and an enlarged reaction area due to near-field interactions around the nanostructured metal surfaces. The numerical simulations using the FDTD method show that the proposed design provides optimal functioning conditions with improved sensitivity.
Wu et al. [63]	Sensitivity improvement is substantial, with a sensitivity of 4720 nm/RIU for gas detection and 5520 nm/RIU for low-refractive-index materials in an aqueous environment.	Enhanced specificity due to the increased adsorption of biomolecules on the graphene surface.	Improved stability, with the graphene ribbon array providing dynamic tunability and consistent performance over a wide range of wavelengths from infrared to THz.	The study presents a novel transmission-type SPR sensor based on a graphene ribbon array for infrared wavelengths. Significant improvement in sensitivity and figure of merit (FOM) compared to traditional metal-based SPR sensors. The sensitivity of the sensor is 4720 nm/RIU for detecting gas (refractive index change from 1.0 to 1.05) and 5520 nm/RIU for low-refractive-index materials in an aqueous environment (refractive index change from 1.30 to 1.35). The dynamic tunability of graphene enables detectable refractive index changes covering a broad wavelength range from infrared to THz. The sensor’s performance is influenced by the Fermi level and the number of graphene layers, which provide guidelines for optimizing sensor design.
Maharana et al. [64]	The imaging sensitivity of the proposed sensor is approximately 50% greater than that of the conventional 2S2G–gold–graphene-based affinity sensor.	Enhanced specificity due to the improved adsorption of biomolecules on the graphene layer.	The graphene layer acts as a passivating layer, preserving the plasmonic properties of silver and enhancing stability by preventing oxidation and corrosion.	The study proposes a low-index dielectric (Teflon)-mediated SPR sensor based on graphene in a dielectric–metal–dielectric (D–M–D) configuration for near-infrared measurements. At a wavelength of 850 nm, the field intensity enhancement factor at the graphene-sensing layer interface for the proposed chalcogenide Ge20Ga5Sb10S65 (2S2G)–teflon–gold–graphene-based sensor is 20% greater than for the 2S2G–gold–graphene-based sensor. The penetration depth of the field into the sensing region for the proposed sensor is 340% greater than for the conventional 2S2G–gold–graphene SPR sensor. The FWHM (full width at half maximum) of the SPR curve for the low loss SP (LLSP) mode is 90% smaller than that of the conventional SP (CSP) resonance sensor, enhancing detection accuracy. The proposed sensor demonstrates high imaging sensitivity and stability, making it suitable for high-throughput assessment of multiple simultaneous molecular interactions.
Islam et al. [65]	Approximately 18% sensitivity improvement compared to conventional SPRB.	Enhanced specificity due to improved adsorption efficiency and field amplification.	Improved stability by integrating graphene, which acts as a passivating layer, preventing oxidation and corrosion of the gold layer.	The incorporation of a periodic array of subwavelength grating on top of a graphene layer significantly enhances the sensitivity of the SPR biosensor. The proposed multilayer grating-graphene SPRB demonstrates improved performance in sensitivity, with approximately 18% enhancement compared to traditional SPRB structures. The sensor’s performance is optimized by adjusting the grating configurations (rectangular, sinusoidal, triangular), grating depth, and volume factor. The sensor shows a larger shift of resonance peak, indicating improved sensitivity. Numerical simulations validate the performance enhancements, showing strong plasmonic resonances at optimized configurations.
Toloue et al. [66]	Not explicitly specified in terms of percentage, but the study indicates a significant enhancement.	Not explicitly specified in terms of percentage.	The text mentions improved stability with the prevention of oxidation by the graphene layer.	The study demonstrates that the incorporation of graphene significantly enhances the sensitivity of SPR-based biosensors compared to conventional sensors. This is primarily due to the higher adsorption efficiency of graphene and a greater change in refractive index near the copper surface.
Vermaet al. [67]	The study indicates that the sensitivity of the proposed SPR biosensor with graphene and an air gap is 2.35 times greater than that of a graphene-based SPR sensor without an air gap.	Not explicitly specified in terms of percentage.	The graphene layer provided protection against the oxidation of the silver layer, improving the stability and performance of the biosensor.	The incorporation of both graphene and an air gap significantly enhances the sensitivity of the SPR biosensor. The optimized structure, with a 23 nm air gap and 53 nm silver layer, shows improved performance due to strong excitation of surface plasmons, resulting in a large change in the refractive index at the sensing medium interface.
Wu et al. [68]	Nearly tenfold improvement in sensitivity compared to the conventional SPR biosensor.	Significant increase in detection accuracy (DA).	The graphene layer enhances the absorption of biomolecules and prevents the oxidation of metals, improving stability.	The incorporation of graphene in the LRSPR biosensor leads to nearly tenfold improvement in sensitivity and a significant increase in detection accuracy. The graphene layer enhances surface plasmons and the absorption of biomolecules, leading to better performance.
Verma et al. [69]	The study indicates that the sensitivity of the proposed SPR biosensor with metamaterial and graphene is significantly higher compared to traditional materials. Specifically, the sensitivity improvement is quantified as follows: At 750 nm: 54.75 degrees/RIU for metamaterial, 41.47 degrees/RIU for graphene, and 40.42 degrees/RIU for conventional SPR. At 850 nm: 52.05 degrees/RIU for metamaterial, 42.71 degrees/RIU for graphene, and 41.66 degrees/RIU for conventional SPR. At 1000 nm: 49.10 degrees/RIU for metamaterial, 43.85 degrees/RIU for graphene, and 43.80 degrees/RIU for conventional SPR.	The detection accuracy (DA) is used as a measure of specificity. The study shows the following: At 750 nm: 5.63/degree for metamaterial, 0.88/degree for graphene, and 0.98/degree for conventional SPR. At 850 nm: 7.34/degree for metamaterial, 1.13/degree for graphene, and 1.22/degree for conventional SPR. At 1000 nm: 17.67/degree for metamaterial, 1.56/degree for graphene, and 1.65/degree for conventional SPR.	The study describes that the incorporation of graphene enhances the stability of the biosensor due to the stable adsorption of carbon-based biomolecules (e.g., ssDNA). Additionally, the metamaterial film further improves the adsorption efficiency, leading to enhanced stability.	The study demonstrates that the incorporation of graphene and metamaterial significantly enhances the performance of the SPR biosensor. The metamaterial-based SPR biosensor exhibits superior sensitivity, detection accuracy, and quality factor compared to graphene-based and conventional SPR biosensors. The enhancement is attributed to the high adsorption efficiency of the metamaterial film and the stable adsorption properties of graphene.
An et al. [70]	The sensitivity of the proposed quasi-D-shaped optical fiber plasmonic biosensor shows a wavelength interrogation sensitivity of 3908–10,693 nm/RIU in the dynamic index range from 1.33 to 1.38. This indicates a significant improvement in sensitivity compared to traditional materials.	The specificity improvement is not explicitly mentioned in terms of percentage, but the study demonstrates a maximum amplitude sensitivity of 95 RIU^−1^ at the wavelength of 2040 nm with the analyte refractive index of 1.37, suggesting an enhanced specificity.	The incorporation of graphene enhances the stability of the biosensor due to the stable adsorption properties of graphene and the ITO layer. This stability is further improved by the design of the quasi-D-shaped fiber, which reduces the likelihood of damage during manufacturing.	The study demonstrates that the incorporation of graphene and ITO layers in the quasi-D-shaped optical fiber plasmonic biosensor significantly enhances the performance. The sensor exhibits a high wavelength interrogation sensitivity of 10,693 nm/RIU and a maximum amplitude sensitivity of 95 RIU^−1^. The quasi-D-shaped design also simplifies the manufacturing process and enhances the impact toughness of the sensor.
Huang et al. [71]	Improved sensitivity by using multiple layers of graphene. The sensitivity values were calculated as 51.0, 16.8, 8.4, 4.43, and 2.12°/RIU for different layers of graphene.	Specificity was enhanced due to the higher detection accuracy of the graphene-based sensor. The study noted that more layers of graphene result in higher detection accuracy.	The stability of the graphene-incorporated biosensor is indicated to be high, with the potential for consistent performance over time due to the strong mechanical and electronic properties of graphene.	The study demonstrated that the incorporation of graphene significantly enhances the sensitivity, specificity, and detection accuracy of SPR-based biosensors compared to traditional materials like gold and silver. The optimal performance was achieved with monolayer graphene, which offered a preferable balance between sensitivity and detection accuracy.
Lin et al. [72]	The maximum intensity sensitivity of the proposed biosensor with graphene is 3.5 × 10^4^/RIU, which is significantly greater than that in the conventional SPR or BSW sensors. For conventional SPR structures, the intensity sensitivity is about 100/RIU.	The specificity improvement is not quantitatively specified, but the study indicates that the proposed graphene-incorporated biosensor shows superior performance in terms of full width at half maximum (FWHM) and detection accuracy compared to conventional SPR sensors.	The study does not explicitly mention stability over time. However, it suggests that the energy losses are within an acceptable range with the graphene layer number varying from 2 to 5, implying stable performance within these parameters.	The novel biosensor configuration consisting of a one-dimensional photonic crystal (1DPC) and graphene as a defect layer significantly enhances the intensity sensitivity. The optimized structure achieves a maximum sensitivity of 3.5 × 10^4^/RIU, outperforming conventional SPR and BSW sensors. The findings emphasize the superior performance in terms of FWHM and detection accuracy with the inclusion of graphene.
Farmani et al. [73]	The study demonstrated that the highest sensitivity is achieved with a monolayer CVD graphene thickness of 0.335 nm and silicon dioxide/silicon thickness of 300 nm at an incident angle of 62.5°.	The use of graphene led to significant improvements in specificity due to the strong coupling condition between the incident light and the graphene–silicon dioxide/silicon structure.	The study indicates that the graphene-incorporated biosensor showed stable performance with low linear and surface defects, verified through atomic force microscopy (AFM) and scanning electron microscopy (SEM).	The study presented a simple, low-cost, compact-footprint, label-free, and high-resolution graphene plasmonic sensor. The optimal performance was achieved with a monolayer CVD graphene thickness of 0.335 nm, silicon dioxide/silicon thickness of 300 nm, and an incident angle of 62.5°. Both theoretical and experimental results verified that the strong coupling condition significantly enhances the resolution and sensitivity of the SPR-based biosensor compared to traditional materials.
Hossain et al. [74]	Total of 21.48% improvement in sensitivity when a 20-V gate voltage is applied to the graphene monolayer compared to no gate voltage.	Enhanced specificity compared to state-of-the-art glucose sensors. The sensor is highly selective to blood sugar levels (BSL).	The sensor shows consistent performance and is less sensitive to environmental variations. The detection error remains within 4.75% on average and within 7.40% in the worst-case scenario when temperature varies by ±10 °C from a reference 25 °C.	The gate-controlled graphene SPR glucose sensor offers significant improvement in detection sensitivity and figure of merit compared to state-of-the-art SPR biosensors. The proposed sensor does not require labels or extensive sample preparation and is highly sensitive and selective to blood sugar levels while being less affected by environmental changes.
Behboudi et al. [75]	The study demonstrated a sensitivity of approximately 1.5 THz/Permittivity unit, which is adequate for sensing purposes at the given frequency range and thickness.	Enhanced specificity is achieved through unique spectral features such as Accumulated Spectral Power (ASP) and Averaged Group Delay (AGD), which are independent of the resonance frequencies and operate over a broad range of the spectrum.	The graphene-based sensor exhibits high stability and consistent performance, able to detect relative permittivity variations up to 4 with a resolution of 0.1 and thickness variations from 5 µm to 600 µm with a resolution of 0.5 µm. These capabilities are considered significantly higher than previously reported works.	The novel THz spectroscopy technique proposed in the study utilizes a graphene-based metasurface that operates in reflection mode. The sensor can detect variations in relative permittivity and thickness with high precision, supported by spectral features like ASP and AGD. The study highlights the superior field confinement and surface plasmonic resonance capabilities of the graphene-based metasurface compared to traditional noble metals such as gold, copper, and platinum.
Mostufa et al. [76]	Achieved a sensitivity of 230.77 deg/RIU, which is higher compared to other configurations (e.g., 140.35 deg/RIU for traditional materials).	Enhanced specificity indicated by high detection accuracy (DA) of 0.161 deg^−1^ and a figure of merits (FOM) of 37.22 RIU^−1^.	Demonstrated consistent and reproducible performance in detecting the SARS-CoV-2 virus, showing reliable results across different concentrations of analytes.	The study showed that the graphene-based multilayered SPR biosensor has significantly higher angular sensitivity (230.77 deg/RIU) and improved performance metrics compared to traditional SPR biosensors. The sensor can effectively detect the presence of the SARS-CoV-2 virus rapidly without false-positive results. The proposed configuration outperformed other existing models, demonstrating superior sensitivity and specificity.
Ishtiak et al. [77]	The proposed sensor achieved a significantly higher value of sensitivity at 397.71 deg./RIU.	Enhanced specificity, achieving a wide range of detection accuracy from 0.199 to 0.498 (1/deg.) with a high-quality factor of 99.50 (1/RIU).	The sensor shows high stability with a considerably low minimum reflectance of 0.05 (normalized) for the entire salinity detection range.	The study presents a graphene-based SPR sensor for rapid water salinity concentration detection. The sensor demonstrated significantly higher sensitivity and specificity, improved stability, and high efficiency in detecting salinity levels from 1% to 30% using a multiple-light source technique.
